# Exploring nature-based art therapy: a scoping review

**DOI:** 10.3389/fpsyg.2025.1522629

**Published:** 2025-01-29

**Authors:** Elīna Gulbe, Aija Ozola, Beāte Vītola, Elīna Akmane, Jasmina Pacek, Kristīne Mārtinsone

**Affiliations:** ^1^Riga Stradins University, Riga, Latvia; ^2^Department of Health Psychology and Paedagogy, Riga Stradins University, Riga, Latvia; ^3^Academy of Arts and Culture in Osijek, Osijek, Croatia

**Keywords:** art therapy, eco-art therapy, nature, nature-based art therapy, scoping review

## Abstract

**Introduction:**

Nature-based art therapy is a therapeutic approach that uses natural elements and settings to promote well-being and health through creative expression, facilitated by a professional art therapist. Interest in nature-based mental health approaches surged, particularly following the COVID-19 lockdowns, as research highlights nature’s role in health restoration and sustainability. Despite growth in the field, a comprehensive overview of nature-based art therapy remains absent. This scoping review aimed to map the research landscape and identify the thematic scope within this emerging field.

**Methods:**

A systematic search following the PRISMA-ScR guidelines across the ProQuest, SAGE, Scopus, Taylor & Francis, and ScienceDirect databases identified 11 publications that address art therapy involving nature and are published in English.

**Results:**

The review identified four key themes: areas of focus, nature engagement, core elements, and challenges in nature-based art therapy. The areas of focus encompassed overall mental health and well-being, emotion regulation and stress management, cognitive development, social bond and support, self-discovery and personal growth, trauma and grief management, creative self-expression, and environmental sustainability. The core elements, such as artwork, materials, and therapy settings, were identified. Although nature-based therapy, involving both direct and indirect nature engagement, described promising benefits, specific challenges, including complexities in client assistance, constraints in nature access, psychosocial and cultural barriers, and risk management, were also highlighted.

**Discussion:**

This scoping review provides a comprehensive framework for understanding nature-based art therapy and underscores the need for further theoretical and practical evidence-based development in this field.

## Introduction

Research widely recognizes nature-based therapeutic practices as accessible and affordable approaches to promote mental, physical, and social well-being (Harper et al., 2021). However, the global need for nature-based experiences enabling individuals to connect with nature despite environmental limitations or health concerns has been particularly underscored since the COVID-19 pandemic outbreak in 2020 (Elkis-Abuhoff et al., 2022). This unprecedented challenge highlighted nature’s invaluable role as a resource for mental health, showcasing the benefits of nature-based solutions in promoting well-being, health restoration, and environmental sustainability (Menhas et al., 2024).

Nature-based interventions refer to planned, purposeful activities aimed at enhancing individuals’ functioning, health, and well-being, or facilitating restoration and recovery through exposure to or interaction with authentic or technologically simulated nature (Gritzka et al., 2020). Rooted in the philosophy and theoretical frameworks of ecopsychology, this field seeks to re-establish human connection with nature, promoting a more sustainable and environmentally conscious society (Rhodes and Dunk, 2023). Interventions such as ecotherapy (Summers and Vivian, 2018), nature therapy (Berger, 2020), forest bathing (Subirana-Malaret et al., 2023), forest therapy (Mazzoleni et al., 2024), and outdoor therapy (Owens and Bunce, 2022), among many others, are gaining increasing recognition by offering professionally facilitated experiences of nature contact and nature-based treatment. In past decades, art therapy has contributed to nature-based interventions incorporating nature-based activities and practically exploring the potential of intersection between arts and nature within the therapeutic process.

According to the American Art Therapy Association, art therapy is a mental health profession that enriches the lives of individuals, families, and communities through active art-making, creative processes, applied psychological theory, and human experience within a psychotherapeutic relationship (American Art Therapy Association, 2022). Recent systematic evidence highlights the broad application of art therapy in alleviating psychiatric symptoms, enhancing psychological well-being, reducing social and behavioral problems, and improving cognitive function and various somatic symptoms (Joschko et al., 2024), as well as treating psychological trauma (Maddox et al., 2024). However, while some countries recognize art therapy as a distinct mental health and human services profession, it is also widely employed as a treatment modality within broader therapeutic contexts, conducted by art therapists.

Used in education, social care, health care, and private practice with diverse client groups, art therapy enables individuals, whether in individual or group sessions, to express, recognize, and transform their emotions, needs, and motivations, to identify and change problematic behavior patterns, and to develop and strengthen specific skills (Mārtinsone and Duhovska, 2023). However, the use of art for health and well-being is not limited to art therapy, as similar elements appear in a range of art-based interventions conducted by other professionals who incorporate art therapy methods to enrich their practices and enhance client outcomes. By contrast, when conducted by specifically trained professionals, art therapy focuses on artistic expression as the primary means of promoting and maintaining well-being through personal growth, stronger interpersonal relationships, enhanced community health, increased self-awareness, and greater resilience. Additional benefits include improved cognitive function, positive social impacts, and contributions to ecological awareness (American Art Therapy Association, 2022). In response to global ecological challenges, art therapists are encouraged to reshape their practices to align with principles of environmental sustainability and to help individuals reconnect with nature on a personal level (Van Lith, 2024).

Nature-based art therapy (NBAT) is a growing field within art therapy. While terminology in this field is diverse and evolving, the umbrella term “nature-based art therapy” encompasses any approach that integrates natural elements within art therapy, emphasizing nature as a key component of the therapeutic process. Thus, NBAT refers to a therapeutic approach that uses natural elements and settings to promote well-being and health through creative expression, facilitated by a professional art therapist.

From an ecological perspective, NBAT envisions an impact on both the individual and the environment. For example, Carpendale (2010) proposes an ecological approach to art therapy, emphasizing interconnectedness and grounding it in ecopsychology principles (Scull, 2008). Eco-art therapy describes this discipline as a blend of ecotherapy and art therapy (Sweeney, 2013), emphasizing both the psychological and ecological benefits of engaging with nature as material and setting (Pike, 2021). Environmental art therapy focuses specifically on the natural environment as a therapeutic setting and incorporates a broader range of arts-based practices, such as movement, sound, and creative expression, highlighting the therapeutic potential of experiencing nature’s rhythms and processes (Heginworth and Nash, 2019). In turn, nature-assisted art therapy that emerged from recognizing the interconnectedness between art, nature, and human sciences, aims to integrate these fields into a holistic approach to well-being (Kopytin, 2021). Finally, forest art therapy is conducted in settings with trees, while outdoor art therapy encompasses any form of art therapy that takes place outdoors, including but not limited to forests and parks (Lee et al., 2020; Wright et al., 2023; Wardle, 2023).

Despite the field’s diverse applications, a comprehensive overview of nature-based art therapy remains lacking, highlighting the need for the current research. Scoping reviews, as outlined by Peters et al. (2024), are designed to systematically map the existing literature and identify gaps in knowledge, making them particularly suited for underexplored and emerging fields. This approach is well-suited to NBAT, where diverse practices and theoretical frameworks require synthesis to provide a holistic understanding. To address this gap, we conducted a scoping review to map the research landscape and identify the thematic scope within this emerging area, addressing the research question: What is the thematic scope in nature-based art therapy?

## Materials and methods

We conducted a scoping review, as it systematically maps existing knowledge on a specific topic and helps to identify key themes and pinpoint areas requiring further research (Munn et al., 2022). We also drew inspiration from previous art therapy research that employed this type of review to explore an art-based therapeutic approach (Finkel and Bat Or, 2020).

Our methodology closely followed the five-stage framework developed by Arksey and O’Malley (2005), which included defining research questions, identifying relevant literature, selecting studies, organizing data, synthesizing results, and incorporating expert consultation. Additionally, we used the Preferred Reporting Items for Systematic reviews and Meta-Analyses extension for Scoping Reviews guidelines (PRISMA-ScR; Tricco et al., 2018) to ensure consistency and rigor in our methodology. PRISMA-ScR checklist is provided in Supplementary Table S1.

### Search strategy

We conducted the first comprehensive search of scientific databases on October 1, 2023, and updated it on January 31, 2024. In collaboration with an academic librarian, we developed a multi-step search strategy and searched databases including ProQuest, SAGE Journals, Scopus, Taylor & Francis, and ScienceDirect. These databases were selected for their broad coverage of scientific sources in the social sciences and healthcare fields, including art therapy and various nature-based interventions.

Our search strategy focused on key terms related to both art therapy and nature. To identify therapeutic practices led by professional art therapists, we used terms such as “art therapy,” “arts therapy,” and “art psychotherapy.” These were combined with nature-related terms like “ecological,” “nature-based,” and “environmental” to reach the relevant sources. Due to limitations on the number of search terms allowed in some databases, we prioritized terms that consistently produced the most relevant results. Using Boolean operators “AND” and “OR” we developed a final search strategy to capture the most pertinent research related to our topic, as detailed in Table 1.

**Table 1:** Search strategy.

Search components	Search strategy
Keywords: art therapy	((“art therapy” OR “arts therapy” OR “art psychotherapy”)
	AND
Keywords: nature based	(“ecological approach” OR “nature based” OR “environmental”))

### Eligibility criteria

We used the systematic review tool Rayyan Systems (2023) to ensure accuracy and comprehensiveness in our selection process. Based on a modified PICOS statement (Methley et al., 2014), we established specific eligibility criteria. We included articles addressing art therapy clients, with no restrictions on age or health conditions, as well as articles featuring perspectives from art therapists (Population). The interventions described needed to be conducted by professional art therapists and align with the concept of art therapy (Intervention). We considered art therapy conducted in nature or incorporating natural elements as relevant (Context). This included therapy using natural elements that were mentally or digitally produced (e.g., nature-related metaphors or nature photographs), even if implemented indoors. No restrictions were set regarding the outcomes of art therapy (Outcome). We included various study designs, including quantitative, qualitative, and mixed-method studies, as well as case studies and perspective articles (Study design). Our search focused exclusively on peer-reviewed publications in scientific journals, with full texts available in English.

We applied exclusion criteria to omit articles that did not specifically reference nature-based art therapy. We excluded other nature-assisted art interventions conducted in psychological counseling, psychotherapy, social work, education, or community programs, led by professionals other than art therapists. Additionally, we excluded articles that referenced nature-based activities in art therapy without directly using natural elements, as defined above, and rather discussed nature-related topics (e.g., climate change). We also excluded studies on nature-based therapies conducted in other arts therapies, such as drama, dance/movement, and music therapy, which should be noted, as the term “art therapy” is often used to encompass several of these modalities. Furthermore, gray literature and certain types of peer-reviewed content, including editorials, comments, book reviews, and conference proceedings, were excluded. The search was not restricted to any specific publication period.

**Figure 1:**
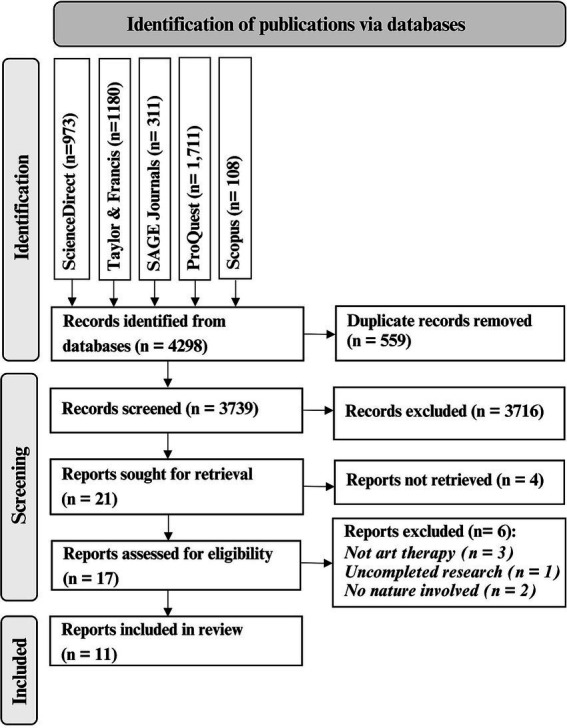
PRISMA flow diagram for scoping review.

Thus, we sourced articles from scientific databases using the specified search strategy. The initial search yielded 3,739 publications after removing 559 duplicates. Following a meticulous evaluation of each source based on our eligibility criteria, we ultimately included 11 articles in this scoping review. The PRISMA flow diagram (Page et al., 2021), shown in Figure 1, outlines the systematic search and screening process.

### Data extraction and charting

To ensure a structured and standardized approach to data extraction, we developed a data charting table in Microsoft Word 2024 based on the PRISMA-ScR guidelines (Tricco et al., 2018). We systematically collected data on various aspects of each article, including authorship, publication year, research country, publication type, measurements, procedures, study population with details on age, disorders or difficulties addressed, setting, focus of the study or article, and main findings. To ensure accuracy and consistency, the first and third author independently charted the data and conducted a peer review of the categorization. Subsequently, an invited researcher charted and verified the data in duplicate. Finally, the validation of the data was completed by the entire research team.

### Data analysis

For data processing and analysis, we conducted a thematic analysis using the seven-step framework outlined by Braun and Clarke (2006). This involved organizing and elaborating on datasets to gain deeper insights into the research topic and to develop a more nuanced understanding of the research data. It went beyond simple description, allowing for the interpretation of various aspects of the subject and enabling adaptation of methods to align with specific research needs and theoretical frameworks (Braun and Clarke, 2006). The systematic process included familiarizing with the data, generating initial codes, searching for themes, reviewing themes, defining and naming themes and subthemes, producing a report, and completing interpretative analysis. Following this framework enabled us to uncover meaningful patterns and insights within the collected data, thereby strengthening our understanding of the research landscape in nature-based art therapy.

## Results

### General characteristics

The selected articles reflected the variety of participant demographics and research methodologies employed in NBAT, as shown in Supplementary Table S2. The majority focused on qualitative research, detailing the therapeutic process (6 articles). Two articles provided theoretical perspectives, while a smaller proportion investigated the effectiveness of nature-based art therapy (3 articles). The findings indicated that this field had been researched over the past three decades, with articles available from various countries. Overall, although relatively small, the research landscape is dynamic, with an increase in publications since 2020, illustrating a growing interest in nature-based art therapy.

The frequency of publications remained relatively consistent, averaging one article every five to ten years since 1992. However, since 2020, the volume of research has grown, with one to three articles published annually. Studies conducted in various countries highlighted the international relevance of NBAT, with research included from the United States, United Kingdom, Israel, and South Korea, as well as perspective articles from Canada and Russia.

Studies involved a diverse range of participants (see Supplementary Table S3), demonstrating the broad applicability of NBAT across populations. Notably, most studies featured small sample sizes. Participants included children (Steinhardt, 1998; Kang et al., 2021; Wardle, 2023), adolescents (Klorer, 1992), and adults (Steinhardt, 1998; Peterson, 2015; Lee et al., 2020; Elkis-Abuhoff et al., 2022; Gavron et al., 2023; Wright et al., 2023), ranging from as young as three years old (Steinhardt, 1998) to 65 years (Elkis-Abuhoff et al., 2022). NBAT clients included adults and children experiencing trauma and grief (Steinhardt, 1998; Wardle, 2023), children experiencing hyperactivity (Steinhardt, 1998), non-disabled siblings of children with disabilities (Kang et al., 2021), physically and emotionally abused adolescents (Klorer, 1992), young adult students navigating life changes (Gavron et al., 2023), and adults facing mental health challenges, such as stress, depression, anxiety, and aggression (Lee et al., 2020; Wright et al., 2023). The inclusion of cancer patients (Peterson, 2015) and individuals affected by lockdown (Elkis-Abuhoff et al., 2022) further highlighted the relevance of NBAT in addressing both personal and societal challenges.

### Identifying the thematic scope

To identify the primary thematic scope, we conducted a thematic analysis (Braun and Clarke, 2006), involving an in-depth immersion in the extracted data. Coding was used to categorize the thematic scope of the selected articles. Each code served as a label assigned to text segments (coding units), capturing the meaning of that unit. The process began with an initial round of coding, in which we labeled relevant coding units aligned with our research question. This was followed by a second round of coding, during which we grouped the codes into themes and organized them in relation to one another (Wæraas, 2022).

In the initial phase, we adopted an inductive, data-driven approach, allowing the data to shape our understanding. However, it should be acknowledged that thematic analysis cannot be entirely inductive, as our existing knowledge and theoretical frameworks inevitably influence our interpretation. Due to the inductive nature of our analysis, the data guided the development of themes, enabling text fragments to be categorized under multiple codes (Wæraas, 2022). After thoroughly reviewing all publications, we categorized 737 text fragments into 86 codes using NVivo software to systematize the data. Through repeated reviews of the articles, we consolidated the initial codes under four overarching themes, resulting in 85 distinct codes. Ultimately, by analyzing these four themes, we grouped the initial codes into common themes and identified relevant subthemes, as detailed in Supplementary Table S3.

Based on thematic analysis, four key themes emerged within the scope of nature-based art therapy: (1) areas of focus, (2) nature engagement, (3) core elements, and (4) challenges. Each theme was defined as shown in Table 2, and the study results were reported according to these identified key themes (see Table 2).

**Table 2:** Key themes in nature-based art therapy.

Key theme	Definition
Areas of focus	Therapeutic directions of nature-based art therapy to enhance mental health and well-being, support personal growth and development, and assist in managing psychosocial challenges or mental health disorders
Nature engagement	Interaction with nature and natural elements within nature-based art therapy, either directly or indirectly, to foster connection, creativity, and self-reflection within the therapeutic process
Core elements	Fundamental components and structures of nature-based art therapy that form the basis of a therapeutic practice, encompassing the types of artwork, materials, and therapy settings that shape how therapy is conducted and facilitate client engagement with nature and artistic expression
Challenges	Ethical, environmental, and practical considerations related to client assistance, nature access, psychosocial and cultural barriers, and risk management which art therapists must address to ensure ethical, safe, and well-conducted therapy when integrating nature-based practices

The complete thematic classification, illustrated with example vignettes, is available in Supplementary Table S3. Along with relevant subthemes, it provides a framework for understanding NBAT and addresses the research question of the current scoping review.

### Areas of focus in nature-based art therapy

The first key theme emerged from the identification of perceived or targeted benefits of the therapeutic process, as reported by clients in the studies, as well as by art therapists and researchers. We agreed on the term “areas of focus” for this heterogeneous theme and defined it as therapeutic directions of nature-based art therapy aimed at enhancing mental health and well-being, supporting personal growth and development, and assisting in managing psychosocial challenges or mental health disorders. The identified subthemes indicated that, by harnessing the therapeutic potential of artistic expression with natural elements, NBAT tends to develop a comprehensive framework for promoting psychological well-being, personal growth, and environmental sustainability. The areas of focus addressed were mental health and well-being, emotion regulation and stress management, cognitive development, social bond and support, self-discovery and personal growth, trauma and grief management, creative self-expression, and environmental sustainability, each represented by several codes as shown in Table 3. These areas are explored in greater detail below.

**Table 3:** Subthemes and codes for the key theme “areas of focus”.

Subthemes	Codes
Mental health	Aggression; anxiety; depression
Mental well-being	Life satisfaction; positive affect
Emotion regulation and stress management	Emotion expression; emotion recognition; fear management and resolution; stress reduction
Cognitive development	Aesthetic awareness; attention and focus; creative thinking; recall of memories; problem solving
Social bond and support	Community building; group support and cohesion; Impact of group interaction; perspective sharing; relationship building skills; trust development
Self-discovery and personal growth	Body–mind-environment awareness; development of ecological identity; development of responsibility; self-esteem enhancement; self-reflection; sense of achievement
Trauma and grief management	Grief and loss management; trauma-informed care
Creative self-expression	Creative potential; flow state; playfulness and enjoyment
Environmental sustainability	Environmental awareness and knowledge; environmentally responsible behavior

Studies with quantitative and mixed methods have demonstrated that nature-based art therapy significantly enhances various aspects of mental health and wellbeing. Kang et al. (2021) implemented a rigorous experimental design with randomization and pre-test/post-test evaluations using EEG and psychological scales. This methodology robustly supports the therapeutic impacts on stress, attention, and self-esteem. Notable improvements were seen in reducing aggression, anxiety, and depression. The incorporation of ecological environments into art therapy was crucial in mitigating these issues, as Lee et al. (2020) documented. Additionally, Elkis-Abuhoff et al. (2022) employed a mixed-method approach, utilizing both the Satisfaction with Life Scale (SWLS) and the Positive Affect and Negative Affect Schedule (PANAS). This allowed for a comprehensive assessment of life satisfaction and emotional states pre- and post-therapy, enhancing the reliability of the findings through quantitative measures and qualitative feedback.

This scoping review also underscored the role of nature-based art therapy in emotion regulation and stress management. Enhancing emotion expression (Klorer, 1992; Lee et al., 2020; Wardle, 2023; Gavron et al., 2023) and recognition (Klorer, 1992; Lee et al., 2020), managing fear (Klorer, 1992; Steinhardt, 1998; Carpendale, 2010; Wright et al., 2023; Wardle, 2023), and reducing stress (Lee et al., 2020; Kang et al., 2021) were often an intention. For example, Wilderness Stress Challenge program (Klorer, 1992) facilitated emotional processing, also art therapy sessions held in nature may have helped in recognizing and expressing emotions to a boy after a loss of his family member (Wardle, 2023). Additionally, stress reduction was observed across various life areas, providing substantial relief to participants (Lee et al., 2020; Kang et al., 2021).

Trauma and grief management within nature-based art therapy (NBAT) specifically addressed the complex emotional landscapes of individuals experiencing loss or trauma. This theme included grief and loss management (Carpendale, 2010; Wardle, 2023), where therapeutic processes focused on life’s cyclic nature, helping individuals to reflect and navigate the stages of grief from loss to transformation. The subtheme trauma-informed care, documented by Klorer (1992), Gavron et al. (2023), and Wardle (2023), highlighted tailored approaches within NBAT that supported individuals in processing traumatic events, particularly in cases of meaningful loss and bereavements.

The theoretical proposal and research in nature-based art therapy has shown the aim of cognitive development. Art therapists and researchers noticed the intention to intensify aesthetic awareness (Gavron et al., 2023; Wright et al., 2023), attention and focus (Carpendale, 2010; Kang et al., 2021), creative thinking (Gavron et al., 2023; Wright et al., 2023). The authors also demonstrated art and nature’s impact on memory recall (Lee et al., 2020; Elkis-Abuhoff et al., 2022; Gavron et al., 2023). Enhancing problem-solving skills (Carpendale, 2010; Kang et al., 2021; Lee et al., 2020; Elkis-Abuhoff et al., 2022; Gavron et al., 2023) as development of other cognitive functions occurred both in individual sessions and in interaction with group members.

Art therapy outdoors often was held in groups, making social bond and group support possible. In NBAT community building is important (Klorer, 1992; Carpendale, 2010; Wright et al., 2023). The open outdoor spaces and group creative processes were highlighted as conducive environments for fostering group support and cohesion (Wright et al., 2023; Gavron et al., 2023). Participants and therapists have noted that supportive group interactions facilitate individual development and the identification and sharing of diverse perspectives (Wright et al., 2023). It is also noted that therapeutic settings of NBAT may strengthen interpersonal relationship building skills (Wright et al., 2023) and build trust among participants in wild outdoor settings (Klorer, 1992).

Creative self-expression in NBAT leveraged the therapeutic power of art to foster creative potential, flow state and playfulness and enjoyment. Enhancing creative potential, where using natural materials in artmaking allowed individuals to express themselves freely without the constraints of traditional artistic skill or technique (Gavron et al., 2023; Wardle, 2023). Authors mentioned playfulness, spontaneity and joy as an important part of NBAT (Kang et al., 2021; Gavron et al., 2023; Wright et al., 2023; Wardle, 2023). This kind of engaging in creative nature-based therapy activities facilitated a mental state of flow, helping individuals achieve deep immersion in their tasks (Gavron et al., 2023). This aspect of creativity leads to the next subtheme. Nature-based art therapy promotes self-discovery and personal growth, focusing on various subareas. The thematic analysis underscored the significance of developing an ecological identity or the sense of belonging to the wider world, advocating an ecological therapeutic approach that aligned individual identities with broader societal and environmental contexts (Klorer, 1992; Carpendale, 2010; Peterson, 2015; Kopytin, 2021; Elkis-Abuhoff et al., 2022; Kang et al., 2021; Wright et al., 2023; Wardle, 2023; Gavron et al., 2023). Ecological identity might have been enhanced by body–mind-environment awareness, as mentioned by Kopytin (2021) and many other authors (Klorer, 1992; Carpendale, 2010; Peterson, 2015; Elkis-Abuhoff et al., 2022; Kang et al., 2021; Wright et al., 2023; Wardle, 2023; Gavron et al., 2023). Research indicated that creative processes with nature materials fostered a sense of achievement (Gavron et al., 2023; Wright et al., 2023) and open outdoor spaces may widen psychological boundaries and may support self-reflection (Peterson, 2015; Lee et al., 2020; Wright et al., 2023; Wardle, 2023). NBAT integrated mindfulness with ecotherapy practices, helping individuals reconnect with their surroundings and themselves (Peterson, 2015; Kopytin, 2021). Moreover, NBAT has been effective in enhancing overall and social self-esteem, with notable improvements documented by Kang et al. (2021). NBAT not only aided in cultivating a sense of belonging but also enhanced responsibility to self and others (Klorer, 1992) and the entire ecosystem we live in (Gavron et al., 2023).

Environmental sustainability in NBAT reflected the dual focus on healing the individual and the environment. This theme unfolds through two main components: environmental awareness and knowledge (Steinhardt, 1998; Carpendale, 2010; Kopytin, 2021) and environmentally responsible behavior (Carpendale, 2010; Kopytin, 2021; Elkis-Abuhoff et al., 2022; Gavron et al., 2023). For example, terrarium building not only teaches participants about ecosystems but also inspires them to view their creations as self-sustaining systems. The responsible behavior might be promoted by the practice of using sustainable materials and encouraged behaviors that contributed positively to environmental health, as seen in the works of Carpendale (2010), Kopytin (2021), Elkis-Abuhoff et al. (2022), and Gavron et al. (2023).

### Nature engagement in nature-based art therapy

As indicated by results, nature engagement is very central within nature-based art therapy. The word “nature” appeared 718 times in eleven included articles, making it the third most common term, trailing only “art” and “therapy,” as detailed in Table 4.

**Table 4:** Word frequency within included articles.

Word	Count	Similar words
Arts	693	Art, arts
Therapy	575	Therapies, therapies’, therapy
Nature	525	Natural, natural’, naturally, nature, nature’
Participants	295	Participant, participants, participants’, participate, participated, participating, participation
Experiences	232	Experience, experience’, experiences, experiment, experimented, experiments
Using	207	Use, used, useful, uses, using
Group	177	Group, grouping, groups, groups’
Outdoor	157	Outdoor, outdoor’, outdoors
Sand	153	Sand, sands
Making	149	Make, makes, making
Terrarium	149	Terrarium, terrariums
Sessions	148	Session, sessions
Environments	143	Environ, environment, environments
Health	142	Health
World	142	World, worldly, worlds
Therapists	140	Therapist, therapists, therapists’
Work’	139	Work, work’, worked, working, works
Creativity	139	‘Creative, creative, creatively, creativity
Study	132	Studied, studies, study, studying
Environmentally	129	Environmental, environmentally
Expressive	129	Express, expressed, expresses, expressing, expression, expressions, expressive, expressiveness
One	126	One, ones
Humans	122	Human, human’, humanities, humanity, humans, humans’
Based	121	Base, based, bases
Life	120	Life
May	119	May
Self	118	Self
Support	115	Support, supported, supporting, supportive, supports
Also	114	Also
materials	114	Material, materials

According to the identified scope, we defined nature engagement as interaction with natural elements within nature-based art therapy, either directly or indirectly, to foster connection, creativity, and self-reflection within the therapeutic process. The codes corresponding to each subtheme are presented in Table 5.

**Table 5:** Subthemes and codes for the key theme “nature engagement”.

Subthemes	Codes
Direct engagement	Crafting with natural materials; found object-driven self-reflection; miniature ecosystem building; nature-focused photography; outdoor mindful activities; planting and caring for plants; production of art materials from nature; sensory interaction with natural elements; wildlife exploration
Indirect engagement	Art-based exploration of ecological identity; art-based exploration of experiences in nature; found object-driven self-reflection; landscape art creation; use and creation of nature-inspired poetry; use of analogies in verbal reflection; use of nature-inspired color palette; use of nature-inspired metaphors; work with nature photography

Two categories of nature engagement emerged: direct and indirect. Direct engagement involves clients interacting both physically and mentally with nature, primarily engaging on a sensory level at least with one of five senses, involving actual nature materials and nature as setting. Indirect engagement weaves elements of nature into the therapeutic process more conceptually, focusing on nature as a subject rather than requiring the physical presence of nature materials or outdoor settings. Each category of nature engagement is illustrated through practical examples in structured thematic analysis of nature-based art therapy presented in Supplementary Table S3. Although this categorization does not exclude the simultaneous use of both types of engagement in a single session, it aids in organizing various examples of the process. This classification serves to illustrate the distinct but complementary ways of how nature can be engaged within the therapeutic process.

Direct nature engagement involved hands-on activities that facilitated a deep, interactive connection with the nature materials and outdoor settings. Crafting with nature materials allowed participants to create all kinds of art, for example, sculptures, using items like branches and leaves and grass (Steinhardt, 1998; Carpendale, 2010; Kopytin, 2021; Kang et al., 2021; Elkis-Abuhoff et al., 2022; Gavron et al., 2023). Found objects in nature prompted self-reflection, whether through art making or simply by observing both naturally occurring and human-made objects (Wardle, 2023; Wright et al., 2023). Miniature ecosystem building involved constructing terrariums (Steinhardt, 1998; Elkis-Abuhoff et al., 2022; Gavron et al., 2023), while nature-focused photography captured moments of engagement with nature (Peterson, 2015; Kang et al., 2021).

These and other outdoor mindful activities included sensory experiences through grounding exercises and walks (Peterson, 2015; Kang et al., 2021; Kopytin, 2021; Wright et al., 2023; Wardle, 2023). Planting and caring for plants taught about life cycles and care within a therapeutic context (Gavron et al., 2023), and the production of art materials from natural elements emphasized sustainability and creative use of natural resources (Kang et al., 2021). Sensory interaction with natural elements and wildlife exploration further deepened participants’ ecological engagement and knowledge, enhancing their appreciation for biodiversity (Steinhardt, 1998; Kang et al., 2021; Kopytin, 2021; Wright et al., 2023; Carpendale, 2010; Peterson, 2015; Wardle, 2023; Wright et al., 2023). These activities collectively fostered a comprehensive and immersive experience in nature, crucial for the therapeutic processes of NBAT.

Indirect nature engagement in art therapy encompasses a range of reflective and interpretative activities designed to deepen participants’ awareness, knowledge and emotional connections to themselves and their environment. For instance, the art-based exploration of ecological identity in NBAT utilizes creative mediums such as map-making. This method encourages participants to consider their place within the wider ecological context, enhancing their relationship with the environment through artistic expression (Carpendale, 2010). Art-based exploration of experiences in nature allowed participants to reflect on their interactions with nature through drawing and journaling, providing insights into their emotional landscape (Klorer, 1992). Landscape art creation tasked participants with recreating their favorite outdoor settings through drawings, fostering a deep, personal connection to specific landscapes (Peterson, 2015; Elkis-Abuhoff et al., 2022).

We also noticed the use of nature-inspired poetry and literature in sessions (Carpendale, 2010; Wright et al., 2023). The use of analogies in verbal reflection drew parallels between human-nature processes and characteristics, enriching the understanding of life’s cycles and interconnectedness (Carpendale, 2010; Wardle, 2023; Wright et al., 2023). Nature-inspired color palettes and metaphors helped to connect with nature on a more symbolic level (Steinhardt, 1998; Elkis-Abuhoff et al., 2022). Work with nature photography involved selecting and reflecting on photographs taken during nature engagements, integrating visual art with therapeutic reflection (Carpendale, 2010; Peterson, 2015). These indirect engagement methods enriched the therapeutic journey, allowing participants to engage with nature even when nature is not accessible physically.

### Core elements of nature-based art therapy

Exploring nature-based art therapy, we identified several core elements that were present in every therapeutic session. We defined these core elements as fundamental components and structures of nature-based art therapy that form the basis of a therapeutic practice, encompassing the types of artwork, materials, and settings that shape how therapy is conducted and facilitate client engagement with nature and artistic expression. These elements fell into three main categories: artwork, materials, and therapy settings. Each category included various subcategories, all supported by studies and practices, and is presented in Table 6.

**Table 6:** Subthemes and codes for the key theme “core elements”.

Subthemes	Codes
Artwork	Digital art; environmental art; literary art; visual art
Materials	Conventional art materials; earth-based materials; found human-made objects; plant-based materials; water-based materials
Therapy settings	Indoor therapy room; back yard; forest; garden; greenhouse; meadow; mountain; park; woodland

Artwork in NBAT involved a variety of forms that each served to engage participants in different aspects of creative expression and connection to nature. Given the emphasis of this study on art therapy, it was anticipated that visual art would be frequently used, allowing participants, for example, to interpret and document their interactions with the outdoor settings (Klorer, 1992; Steinhardt, 1998; Carpendale, 2010; Peterson, 2015; Kang et al., 2021; Elkis-Abuhoff et al., 2022; Wright et al., 2023; Wardle, 2023; Gavron et al., 2023). Digital art, as referenced by Peterson (2015) and Kang et al. (2021), included activities where participants photographed elements they found both pleasant and unpleasant, thus exploring personal aesthetic and emotional responses. Environmental art engaged participants in constructing physical installations using nature and recycled materials, fostering a hands-on connection with nature (Kang et al., 2021; Gavron et al., 2023). Literary art utilized narrative and poetic forms to deepen therapeutic experiences, combining mindfulness with artistic expression (Carpendale, 2010; Wright et al., 2023).

Materials used in NBAT were diverse, ranging from conventional to more innovative, environmentally integrated substances. Conventional art materials like markers and pencils were utilized to provide a familiar medium for expression (Elkis-Abuhoff et al., 2022). In a qualitative context, Gavron et al. (2023) utilized semi-structured in-depth interviews and thematic analysis to explore the subjective experiences of college students engaged in making expressive terrariums. This methodological choice provides deep insights into the psychological and emotional processes that occur during NBAT, emphasizing the transformative potential of creative engagement with nature materials. Earth-based materials (Steinhardt, 1998; Carpendale, 2010; Kang et al., 2021; Elkis-Abuhoff et al., 2022; Gavron et al., 2023; Wright et al., 2023) like sand and pebbles, and plant-based materials (Kang et al., 2021; Kopytin, 2021; Elkis-Abuhoff et al., 2022; Gavron et al., 2023; Wardle, 2023) like flower petals and living plants encouraged interaction with nature, fostering ecological awareness and a sensory connection to the environment. Found human-made objects like bottle caps (Wardle, 2023) and water-based materials, including snow (Steinhardt, 1998; Carpendale, 2010), were also used to create art that reflected environmental themes and challenges.

Therapy settings in NBAT varied widely, each setting providing a unique backdrop that influenced the therapeutic process (see Supplementary Table S3). From indoor therapy rooms that brought natural elements indoors (Gavron et al., 2023) to outdoor settings like backyards, forests, gardens, and parks, each location offered different sensory experiences and opportunities for environmental interaction (Peterson, 2015; Kang et al., 2021; Kopytin, 2021; Gavron et al., 2023; Wardle, 2023). These settings were chosen to enhance the therapeutic objectives, whether it was fostering a deep appreciation for nature, facilitating personal or communal activities, or simply providing a peaceful space for reflection and creation. Lee et al. (2020) combined quantitative assessments with qualitative interviews to compare art therapy in different settings, enhancing the understanding of how environmental integration amplifies therapeutic benefits.

### Challenges in nature-based art therapy

Alongside the positive effects of therapy recognized in the articles, research also indicated challenges that art therapists needed to navigate in organizing the therapeutic process and achieving desired outcomes. In line with the identified scope, we defined the challenges in NBAT as ethical, environmental, and practical considerations related to client assistance, nature access, psychosocial and cultural barriers, and risk management which art therapists must address to ensure ethical, safe, and well-conducted therapy when integrating nature-based practices. Four subthemes characterizing these challenges emerged: complexities in client assistance, constraints in nature access, psychosocial and cultural barriers, and risk management. The codes of each subtheme are presented in Table 7.

**Table 7:** Subthemes and codes for the key theme “challenges”.

Subthemes	Codes
Complexities in client assistance	Session planning; client engagement; group dynamics; location and schedule management; privacy management
Constraints in nature access	Budget constraints; institution-related constraints; time and travel constraints; physical mobility-related constraints; urban nature access constraints
Psychosocial and cultural barriers	Multicultural perspectives on nature engagement; levels of nature connection; eco-anxiety and environmental grief
Risk management	Emergency preparedness; equipment requirements; management of physical comfort and needs; outdoor safety training; unpredictable or inclement weather

Complexities in client assistance included the importance of planning session structures to reduce anxiety and ensure safe and productive client engagement (Wright et al., 2023). Managing group dynamics in nature posed challenges, necessitating strategies to balance interaction and maintain a therapeutic environment, as well as logistical coordination of start times and locations in open spaces (Wright et al., 2023). Ensuring confidentiality and client privacy in nature was also a concern, requiring creative solutions to balance privacy with therapeutic benefits (Wardle, 2023).

Research revealed that nature access, though central to NBAT, faced barriers such as institution-related constraints in settings like prisons and medical facilities, where access to outdoor environments was limited by internal safety rules (Elkis-Abuhoff et al., 2022). Budget constraints restricted the frequency of nature-based sessions, making consistent integration of nature into therapy challenging (Wardle, 2023). Travel to and from outdoor settings before, after, or during sessions posed feasibility issues (Lee et al., 2020; Kang et al., 2021; Wright et al., 2023; Wardle, 2023), as did physical mobility-related constraints, which required specific adjustments, such as arranging special transportation (Wardle, 2023) or offering indirect nature engagement indoors (Elkis-Abuhoff et al., 2022). Access to urban nature was constrained during the COVID-19 lockdowns in 2020, with individuals unable to reach natural environments based on their location (Elkis-Abuhoff et al., 2022).

Potential psychosocial and cultural barriers included the need to consider multicultural perspectives on nature engagement and cultural differences in how individuals relate to nature to ensure therapy is accessible and relevant for all clients (Carpendale, 2010; Kopytin, 2021; Elkis-Abuhoff et al., 2022; Gavron et al., 2023; Wardle, 2023). Levels of connection to nature can vary, potentially influencing both engagement in the therapeutic process and outcomes, with stronger connections generally considered to yield greater benefits (Carpendale, 2010; Peterson, 2015; Lee et al., 2020; Kopytin, 2021; Elkis-Abuhoff et al., 2022; Gavron et al., 2023; Wright et al., 2023). Clients’ emotional responses to environmental issues, such as eco-anxiety or environmental despair—including feelings of guilt, fear, and grief—were also highlighted as concerns, underscoring the need for art therapists to be prepared and receptive to these themes (Carpendale, 2010).

Risk management issues included emergency preparedness, such as ensuring emergency contacts and first aid were available during sessions to handle any incidents (Wardle, 2023), and addressing physical comfort and needs, given the lack of basic amenities like shelters and toilets at therapy locations, which required pre-session checks and additional planning (Wardle, 2023). Basic forest safety training was also recommended prior to therapy (Kang et al., 2021). Safety preparedness extended to outdoor equipment requirements to adapt to changing weather conditions and maintain safety and comfort (Wardle, 2023). Finally, unpredictable or inclement weather was considered a challenge for scheduling and conducting sessions, affecting the consistency and planning of NBAT (Kang et al., 2021; Wright et al., 2023; Wardle, 2023).

## Discussion

This scoping review aimed to map the research landscape of nature-based art therapy and identify emerging themes within this therapeutic approach. This discussion focuses on the key findings related to areas of focus, nature engagement, core elements, and challenges of nature-based art therapy as the identified key themes.

The areas of focus, including cognitive development, creative self-expression, emotion regulation and stress management, self-discovery and personal growth, social bonding and support, trauma and grief management, and overall mental health and well-being, have been addressed.

Continued exploration of diverse therapeutic interventions, such as art outdoors, can provide deeper insights into how different nature-based activities affect human health and well-being. Future practices could benefit from wider implementation in clinical settings to enhance therapeutic outcomes across diverse populations. NBAT has shown its focus in enhancing emotional regulation and managing stress. Studies showing NBAT’s role in improving cognitive functions such as attention, memory recall, and problem-solving suggest a need for integrating these practices into educational and rehabilitation settings to leverage their cognitive benefits. The ability of NBAT to foster social connections and support among group members highlights its potential for community-based programs. The role of NBAT in promoting self-discovery and personal growth underscores the importance of personalized therapy approaches. Building emotional connections with nature can enhance self-esteem by fostering a deeper sense of belonging to something greater, beautiful, and powerful. In art therapy, these connections are nurtured through interventions that help individuals explore and express their personal feelings toward the natural world (Steinhardt, 1998; Carpendale, 2010; Gavron et al., 2023) and may encourage clients to explore their relationship with nature, fostering an ecological identity.

NBAT’s contribution to environmental sustainability demonstrates the reciprocal relationship between individual well-being and ecological health. Future practices should emphasize sustainable practices and educate participants on environmental responsibility to reinforce this connection. We suggest that whether participants are invited to create a specific piece of art or engaged in a sensory-based experience, the overarching goal remains the same: to foster a connection with nature. This connection not only encourages reflection on ecological identity and the interrelatedness of all things but also may inspire responsible environmental actions both within art therapy sessions and, most importantly, in daily habits (Bratman et al., 2019; Berger, 2020).

The therapeutic impact of both direct and indirect nature engagements in nature-based art therapy is profound. Studies comparing the effects of direct versus indirect engagement in varied ecological and cultural settings could provide deeper insights into their adaptability and effectiveness. Different examples of activities demonstrate how direct physical interaction with nature can provide substantial therapeutic benefits, promoting ecological identity and a deeper, personal connection with the nature. Conversely, indirect engagement offers a reflective and interpretative form of interaction that is particularly beneficial when direct contact with nature is not feasible. Through methods like art-based exploration of ecological identity, creation of landscape art, and the use of nature-inspired elements in artistic expression, this form of engagement deepens the understanding of one’s relationship with the nature and encourages introspection. Indirect engagement techniques, such as drawing a landscape from nature, employing nature-themed metaphors and analogies, enable symbolic connections with nature, enhancing mental and emotional well-being even for those who are not able to experience nature physically.

We suggest that regular client feedback mechanisms should be implemented to adapt therapy sessions based on clients’ experiences of nature, for example, Lee et al. (2020) combined quantitative assessments with qualitative interviews to compare art therapy in different settings, enhancing the understanding of how direct environmental integration amplifies therapeutic benefits. We propose other researchers exploring more indoor alternatives, maybe considering making a virtual nature-based session using nature videos, sounds, or imagery. For example, observing nature through a window could be considered direct nature engagement. With an open window, one can directly experience the living outdoor setting: feeling the wind, smelling the air, sensing temperature changes, and hearing sounds. Even with the window closed, the tangible presence of nature remains accessible as a vivid or calm sight to observe and as a source for personal reflection, as discussed in the interview with an art therapist by Kopytin (2023).

In our exploration of nature-based art therapy, we delineated several key elements that demonstrate the diversity and depth of practices in this field. Our findings, categorized into artwork, materials, and therapy settings, reveal a rich tapestry of methods that facilitate deep connections between participants and the natural world. The rich array of artwork forms, materials, and settings not only supported varied therapeutic goals but also catered to the diverse needs and preferences of participants, underscoring the flexibility and depth of nature-based art therapy.

The diversity of artwork in NBAT, from drawings and collages to three-dimensional environmental objects, terrariums with living plants to literary forms links creative expression with connection to self, others, and nature. The use of diverse materials, ranging from conventional art supplies to natural and recycled elements, enriches the sensory experiences. NBAT offers nature experience in a variety of therapy settings from indoor spaces to expansive outdoor areas. It is recommended to conduct comparative studies across different settings to systematically assess how environmental variables and different interventions influence therapeutic outcomes.

Therapy carried in outdoor settings offers a unique opportunity to cultivate deep connections with oneself, others, and nature, fostering physical and emotional bonds, responsible attitudes, and sustainable lifestyles that align with the Compendium of World Health Organization (WHO) and other United Nations (UN) guidance on health and environment (World Health Organization, 2022). NBAT may help clients to understand how their actions impact the environment and how a connection with nature can contribute to well-being (Thoma et al., 2021). Ongoing adaptation and refinement of practices, informed by systematic feedback and new ecological insights, could further enhance its effectiveness and scope.

The awareness of challenges, which also involve the risks and limitations of NBAT, might be the initial step toward enhancing therapy’s effectiveness and expanding the safe and justified applicability of nature-based solutions (Sterckx et al., 2024). Continued research and discussion in these areas are essential to refine methodologies and enhance the therapeutic potential of art therapy in and with nature. We suggest it is important to advocate for policies that facilitate access to outdoor settings and integrate NBAT into public health programs, however the exploration of challenges within the context of NBAT underscored the complexity of delivering therapeutic interventions in outdoor settings. To overcome these barriers, it is imperative for practitioners to employ flexible, innovative, and culturally sensitive approaches. Tailoring interventions to reflect the diverse environmental, cultural, and personal backgrounds of participants may ensure the therapy’s relevance and accessibility.

It is critical to understand and address psychosocial and cultural barriers to develop inclusive nature-based art therapy programs that respect and utilize individual differences in environmental interaction. Additionally, exploring NBAT’s potential to address broader health challenges like eco-anxiety, grief, and trauma is necessary. Developing comprehensive resources, such as best-practice manuals and guides for therapists, may enhance the management of sessions in outdoor and diverse cultural settings. We fully agree with implications from previous research that specialized training for art therapists can enhance NBAT’s effectiveness by promoting sustainable practices (Kopytin, 2021). Fostering an ecological identity can help students recognize local green spaces as vital sources of health and well-being, encouraging a renewed connection with nature that they can extend to art therapy participants. This training may also empower art therapists to adopt sustainable lifestyles, both personally and professionally, by using eco-friendly, recycled, and locally sourced materials, emphasizing sustainability in workspace design, and promoting the responsible use of resources (Du Plessis and Postlewaight, 2024). Without integrating nature-based art therapy into curricula or providing specialized professional development, many prospective and practicing art therapists may perceive nature-based practices as risky and unsafe.

In terms of research, the scarce evidence base for nature-based art therapy should be acknowledged. However, we believe that this scoping review can inspire further research and encourage conducting studies in close collaboration between art therapy practitioners and academic researchers. Art therapy students could also be a key target audience, as they can be effectively involved in testing nature-based art therapy interventions and providing professionally grounded feedback on their experiences. To strengthen the scientific rigor and reliability of results, the use of psychometrically validated instruments from the field of psychology is recommended for pre- and post-tests of quantitative or mixed-method studies in nature-based art therapy.

## Limitations of research

It is important to acknowledge certain limitations of this study. The authors acknowledge focusing on English-language publications. While understandable, the review would be more impactful if it included a broader range of literature from diverse languages, to capture the global context of nature-based art therapy. Books and other gray literature were excluded from the review. Given the substantial number of books published in this field, it can be assumed that including gray literature, such as books and dissertations, could provide additional information to enrich the initial framework of nature-based art therapy established in this scoping review. The growing interest in this field suggests that new relevant publications may have emerged since the completion of the scoping review.

As previously noted, interpretation of results derived from thematic analysis largely depends on the researchers’ perspective. Consequently, although the classification agreed in our research team, it is possible that different researchers analyzing the same data might develop an alternative classification. For example, in the key theme of core elements, we identified codes related to artwork, materials, and therapy settings. However, subthemes related to client or therapist factors, or their relationship, could potentially be identified as well. Moreover, further research is needed to more thoroughly elaborate on the concepts related to nature-based art therapy identified through thematic analysis.

## Conclusion

This scoping review mapped the research landscape and identified the thematic scope of nature-based art therapy, revealing four key themes: areas of focus, nature engagement, core elements, and challenges. The data analysis and interpretation established an initial framework for each of these key themes.

First, the research indicated that nature-based art therapy is applied to address various areas of focus, including enhancing mental health and well-being, facilitating emotion regulation and stress management, fostering cognitive development, strengthening social bonds and support, promoting self-discovery and personal growth, managing trauma and grief, encouraging creative self-expression, and advancing environmental sustainability.

Second, the results demonstrated that nature-based art therapy enables both direct and indirect engagement with nature, offering various examples from the therapeutic process on how to connect with nature, oneself, and others through sensory experiences, creative expression, and reflection. According to the findings, nature experiences can be made accessible to almost everyone through sensory, creative, interactive, or reflective activities that provide either direct or indirect engagement with nature.

Third, the study identified the core elements of nature-based therapeutic practice. Artworks related to digital, environmental, literary, and visual art, along with various materials such as conventional art materials, natural elements, and even recycled objects, facilitated connection with nature. Therapy settings ranged from indoor rooms to diverse outdoor settings like forests, gardens, and parks, each offering unique opportunities for creative activities and therapeutic experiences.

Fourth, the challenges faced by clients and art therapists in conducting nature-based art therapy were identified, including complexities in client assistance, constraints in nature access, psychosocial and cultural barriers, and risk management. Addressing these issues requires careful planning and adaptive strategies to optimize therapeutic potential and ensure both psychological and physical safety for clients.

In summary, the future of nature-based art therapy holds significant promise for enriching therapeutic practice within art therapy, especially as individuals seek to reconnect with nature, themselves, and others. This approach offers unique opportunities to address growing mental health and environmental challenges. This scoping review provided a comprehensive overview of the expanding field, highlighting the potential of nature-based art therapy to promote health, well-being, environmental awareness, and sustainability. However, further research is necessary to establish its initial effectiveness. Well-conducted studies, preferably randomized controlled trials with larger sample sizes, are needed to yield more robust and generalizable results.

## Data Availability

The original contributions presented in the study are included in the article/Supplementary material, further inquiries can be directed to the corresponding author.
