# Supplementary material for: Exploring nature-based art therapy: a scoping review
Source: Front Psychol. 2025 Jan 29;16:1522629. doi: 10.3389/fpsyg.2025.1522629 (PMC11814171; doi:10.3389/fpsyg.2025.1522629)
Supplement: Supplementary file 1 [file Supplementary.pdf]

Supplementary Material

Table S1

PRISMA-ScR Checklist

SECTION	ITEM	PRISMA-ScR CHECKLIST ITEM	REPORTED ON PAGE #
**TITLE**			
Title	1	Identify the report as a scoping review.	Click here to enter text.
**ABSTRACT**			
Structured summary	2	Provide a structured summary that includes (as applicable): background, objectives, eligibility criteria, sources of evidence, charting methods, results, and conclusions that relate to the review questions and objectives.	Click here to enter text.
**INTRODUCTION**			
Rationale	3	Describe the rationale for the review in the context of what is already known. Explain why the review questions/objectives lend themselves to a scoping review approach.	Click here to enter text.
Objectives	4	Provide an explicit statement of the questions and objectives being addressed with reference to their key elements (e.g., population or participants, concepts, and context) or other relevant key elements used to conceptualize the review questions and/or objectives.	Click here to enter text.
**METHODS**			
Protocol and registration	5	Indicate whether a review protocol exists; state if and where it can be accessed (e.g., a Web address); and if available, provide registration information, including the registration number.	Click here to enter text.
Eligibility criteria	6	Specify characteristics of the sources of evidence used as eligibility criteria (e.g., years considered, language, and publication status), and provide a rationale.	Click here to enter text.

Information sources*	7	Describe all information sources in the search (e.g., databases with dates of coverage and contact with authors to identify additional sources), as well as the date the most recent search was executed.	Click here to enter text.
Search	8	Present the full electronic search strategy for at least 1 database, including any limits used, such that it could be repeated.	Click here to enter text.
Selection of sources of evidence†	9	State the process for selecting sources of evidence (i.e., screening and eligibility) included in the scoping review.	Click here to enter text.
Data charting process‡	10	Describe the methods of charting data from the included sources of evidence (e.g., calibrated forms or forms that have been tested by the team before their use, and whether data charting was done independently or in duplicate) and any processes for obtaining and confirming data from investigators.	Click here to enter text.
Data items	11	List and define all variables for which data were sought and any assumptions and simplifications made.	Click here to enter text.
Critical appraisal of individual sources of evidence§	12	If done, provide a rationale for conducting a critical appraisal of included sources of evidence; describe the methods used and how this information was used in any data synthesis (if appropriate).	Click here to enter text.
Synthesis of results	13	Describe the methods of handling and summarizing the data that were charted.	Click here to enter text.
**RESULTS**			
Selection of sources of evidence	14	Give numbers of sources of evidence screened, assessed for eligibility, and included in the review, with reasons for exclusions at each stage, ideally using a flow diagram.	Click here to enter text.
Characteristics of sources of evidence	15	For each source of evidence, present characteristics for which data were charted and provide the citations.	Click here to enter text.
Critical appraisal within sources of evidence	16	If done, present data on critical appraisal of included sources of evidence (see item 12).	Click here to enter text.
Results of individual sources of evidence	17	For each included source of evidence, present the relevant data that were charted that relate to the review questions and objectives.	Click here to enter text.
Synthesis of results	18	Summarize and/or present the charting results as they relate to the review questions and objectives.	Click here to enter text.
**DISCUSSION**			

Summary of evidence	19	Summarize the main results (including an overview of concepts, themes, and types of evidence available), link to the review questions and objectives, and consider the relevance to key groups.	Click here to enter text.
Limitations	20	Discuss the limitations of the scoping review process.	Click here to enter text.
Conclusions	21	Provide a general interpretation of the results with respect to the review questions and objectives, as well as potential implications and/or next steps.	Click here to enter text.
**FUNDING**			
Funding	22	Describe sources of funding for the included sources of evidence, as well as sources of funding for the scoping review. Describe the role of the funders of the scoping review.	Click here to enter text.

JBI = Joanna Briggs Institute; PRISMA-ScR = Preferred Reporting Items for Systematic reviews and Meta-Analyses extension for Scoping Reviews.
* Where *sources of evidence* (see second footnote) are compiled from, such as bibliographic databases, social media platforms, and Web sites.
† A more inclusive/heterogeneous term used to account for the different types of evidence or data sources (e.g., quantitative and/or qualitative research, expert opinion, and policy documents) that may be eligible in a scoping review as opposed to only studies. This is not to be confused with *information sources* (see first footnote).
‡ The frameworks by Arksey and O'Malley (6) and Levac and colleagues (7) and the JBI guidance (4, 5) refer to the process of data extraction in a scoping review as data charting.
§ The process of systematically examining research evidence to assess its validity, results, and relevance before using it to inform a decision. This term is used for items 12 and 19 instead of "risk of bias" (which is more applicable to systematic reviews of interventions) to include and acknowledge the various sources of evidence that may be used in a scoping review (e.g., quantitative and/or qualitative research, expert opinion, and policy document).

From: Tricco AC, Lillie E, Zarin W, O'Brien KK, Colquhoun H, Levac D, et al. PRISMA Extension for Scoping Reviews (PRISMAScR): Checklist and Explanation. Ann Intern Med. 2018;169:467–473. doi: 10.7326/M18-0850.

Table S2

Overview of 11 articles included in the scoping review

Author(s)/ Year/ Research Country/ Article Type	Measurements	Procedure	Population (age in years)	Disorder/ Difficulties	Setting	The focus of study/ publication	Main findings
**Carpendale 2010** CANADA Perspective	n/a (not applicable)	n/a	n/a	n/a	n/a	To explore art therapy's contributions to addressing global environmental issues	Qualitative: An ecological art therapy approach uses nature's healing powers to foster deeper connection, enhance self-awareness, and promote inner balance.
**Elkis-Abuhoff et al. 2022** USA Original Research	Satisfaction with Life Scale (SWLS); Positive Affect, Negative Affect Schedule (PANAS)	Two sets of data collection within two phases	59 adults (18 to 65)	Restrictions to their autonomy	Indoors (studio and online)	To evaluate if engaging in a nature-based art therapy session would increase one's life satisfaction and positive affect	Quantitative: improved life satisfaction and positive affect. Qualitative: enabled visual exploration of nature despite autonomy restrictions.
**Gavron et al. 2023** ISRAEL Original Research	Semi-structured in-depth interviews; thematic analysis	Preliminary qualitative study	23 college students (23 to 35)	Personal changes in life	Indoors (greenhouse in college area and home)	To explore the meanings and effects of making and tending an expressive terrarium, as well as its potential as an intervention tool in creative arts therapies. To examine the subjective experiences and perceptions of college students.	Qualitative: The building phase - facilitate an experience of flow, emotional expression, cognitive skills. The tending phase - building a relationship with the growing and changing terrarium. The terrarium - promoting self-observation.

Author(s)/ Year/ Research Country/ Article Type	Measurements	Procedure	Population (age in years)	Disorder/ Difficulties	Setting	The focus of study/ publication	Main findings
**Kang et al. 2021** SOUTH KOREA Original Research	Electroencephalogram (EEG): Attention Quotient (ATQ), Anti-Stress Quotient (ASQ); Stress Scale; Self-Esteem Scale	Simple randomization into experimental and control groups. Pre-test and post-test	29 children (7 to 13)	Non-disabled siblings of children with disabilities	Mountain	To examine changes in brain waves, stress, and self-esteem after a continuous eight-week nature-based art therapy program in the forest in non-disabled siblings of children with disabilities	Quantitative: Total stress score significantly decreased. Immune system strengthened. Positive effect on self-esteem. Qualitative: Somatosensory experience, creative expression, and self-motivation in art therapy with forest activities can elicit positive physical and psychological changes.
**Klorer 1992** USA Case Study	n/a	After writing and drawing in the journal, entries were shared	Adolescents (age not indicated)	Physically and emotionally abused adolescents	Wilderness with trees, caves, cliffs, rocks	1. Development of trust in others; 2. Enhancement of self-esteem; 3. Empowerment; 4. Development of sense of responsibility; 5. Development of group cohesiveness	Qualitative: In their journals, adolescents depict themselves facing fears with strength, showing genuine empowerment and a desire to overcome—an inspiring shift for them.
**Kopytin 2021** RUSSIA Perspective	n/a	n/a	n/a	n/a	n/a	To examine the key presumptions and theoretical foundation of nature-assisted ecological creative arts therapies, a branch of contemporary ecotherapy	Qualitative: Nature-assisted or ecological arts therapies can play a greater role in helping our clients and societies to survive, be healthy, and form an affirming response to the constraints in environmental and natural resources.
**Lee et al. 2020** SOUTH KOREA	Symptom Checklist-90-Revision (SCL-90-R)	Quantitative comparison test, interviews	16 adults (28 to 53)	Stress, depression, anxiety, and aggression	Forest	To improve treatment effects for participants through a new venture called art psychotherapy in the outdoor natural environment	Quantitative: Forest-art therapy improved stress scale scores, demonstrating the effectiveness of using the forest environment in art therapy for self-regulation and reducing stress

Author(s)/ Year/ Research Country/ Article Type	Measurements	Procedure	Population (age in years)	Disorder/ Difficulties	Setting	The focus of study/ publication	Main findings
Original Research							vulnerability and interpersonal sensitivity. Integrating nature into art therapy can significantly enhance mental health, addressing stress, depression, anxiety, and aggression.
**Peterson 2015** USA Brief Research Report	n/a	The therapy process was observed and described by an art therapist	74 adults with cancer and their care partners (age not indicated)	Cancer	Back yard	To enhance wellness, self-awareness, and autonomy in cancer patients and caregivers through a creative, mindfulness-based stress reduction art therapy approach	Qualitative: Walkabout, integrating art therapy and mindfulness, fosters meaning-making, fluidity of experience, and enhanced wellness through outdoor engagement.
**Steinhardt 1998** ISRAEL Brief Research Report	n/a	The therapy process was observed and analyzed by an art therapist	1 girl (3); 1 boy 6; 1 girl (11); 2 women (40)	Traumatic experiences; grief; hyperactivity; poor self-image; low academic motivation; mental health problems; fearful hallucinations	Indoor therapy room	To explore the visual and tactile aspects of sand and water, analyzing the symbolic meaning of forms, their connection to client development and progress, and the historical context within art therapy	Qualitative: Expressive sandplay in therapy is like creating an embodied image in art therapy. Sandworlds develop independently but contribute to the client's overall healing.

Author(s)/ Year/ Research Country/ Article Type	Measurements	Procedure	Population (age in years)	Disorder/ Difficulties	Setting	The focus of study/ publication	Main findings
**Wardle 2023** UK Case Study	n/a	The therapy process was observed and analyzed by art therapist	1 boy (6)	Traumatic experiences and grief	Woodland, meadow, garden	Alex's art therapy sessions aimed to address both trauma and grief	Qualitative: Nature-based art therapy helped express grief, facilitating family memorial rituals. Further research needed to explore its benefits and limitations for diverse populations.
**Wright et al. 2023** UK Original Research	n/a	Semi-structured interview; thematic analysis	4 adults with mental health problems (age not indicated), 3 arts therapists	Mental health problems	Park	To understand service users and therapist experiences of an outdoor arts therapies group, their views on its benefits and challenges	Qualitative: Outdoor settings fostered group cohesion, positive mood, and inclusive creativity, supporting the therapeutic potential of outdoor arts therapy.

Table S3

Thematic analysis: key themes of nature-based art therapy

Themes / subthemes / codes	References	Files count	Text unit count	Example vignettes
Areas of focus within nature-based art therapy		11	187	
Mental health		1	3	
Aggression	Lee et al., 2020	1	1	"Integrating the ecological environment into art psychotherapy can play a vital role in improving mental health, such as stress, depression, anxiety, and aggression" (Lee et al., 2020)
Anxiety	Lee et al., 2020	1	1	
Depression	Lee et al., 2020	1	1	
Mental well-being		1	3	
Life satisfaction	Elkis-Abuhoff et al., 2022	1	2	"Overall, the data showed a positive shift in one's view of their life satisfaction following the intervention" (Elkis-Abuhoff et al., 2022)
Positive affect	Elkis-Abuhoff et al., 2022	1	1	"… the positive affects of urban residents were greatly increased by the nature-based art therapy exercise" (Elkis-Abuhoff et al., 2022)
Emotion regulation and stress management		6	6	
Emotion expression	Klorer, 1992; Lee et al., 2020; Wardle, 2023; Gavron et al., 2023	4	5	"The Wilderness Stress Challenge Program, now in its ninth year, is unique in the intensity of its three-month summer program, and in its use of art therapy as a daily component to help the adolescents process and communicate their feelings" (Klorer, 1992)<br>"Case examples present ways in which moving sessions outdoors may have helped him with exploring and expressing his experience of the death of his relative" (Wardle, 2023)

Themes / subthemes / codes	References	Files count	Text unit count	Example vignettes
Emotion recognition	Klorer, 1992; Lee et al., 2020	2	3	"... to promote introspection and to concretize the feelings associated with the events" (Klorer, 1992)
Fear management and resolution	Klorer, 1992; Steinhardt, 1998; Carpendale, 2010; Wright et al., 2023; Wardle, 2023	5	14	"All participants at some point during the summer, have to confront their fears and self-imposed boundaries. It may be a fear of caves, a fear of the dark, a fear of heights, fear of animals and unknown night creatures, real fears and fears of the imagination" (Klorer, 1992)
Stress reduction	Lee et al., 2020; Kang et al. 2021	2	5	"In addition, the stress scale of the experimental group showed a significant change in all sub-categories: parents ($t = 2.226$, $p < 0.05$), family ($t = 2.941$, $p < 0.05$), friends ($t = 2.460$, $p < 0.05$), study ($t = 2.609$, $p < 0.05$), and school ($t = 2.676$, $p < 0.05$)" (Kang et al., 2021)<br>"In the quantitative comparison test of metal health for the outdoor art-therapy that was held in 2016 and the forest-art therapy program that actively used the ecological environment in 2017, forest-art therapy shower better scores on the stress scale" (Lee et al., 2020)
Cognitive development		7	19	
Aesthetic awareness	Gavron et al., 2023; Wright et al., 2023	2	2	"… can help develop an aesthetic perception that may prompt a change in the emotional experience" (Gavron et al., 2023)
Attention and focus	Carpendale, 2010; Kang et al., 2021	2	2	"For psychological relaxation and to strengthen concentration; To relieve stress and strengthen attention span; To strengthen attention span and concentration" (Kang et al., 2021)
Creative thinking	Gavron et al., 2023; Wright et al., 2023	2	5	"Gathering natural materials and making a collective artwork gave members an experience of play in the sense of open ended creating, a process that supports openness to new ideas and softens rigid thinking" (Wright et al., 2023)
Recall of memories	Lee et al., 2020; Elkis-Abuhoff et al., 2022; Gavron et al., 2023	3	4	"... emotional projects and positive memories were recalled through contact with natural objects" (Lee et al., 2020)

Themes / subthemes / codes	References	Files count	Text unit count	Example vignettes
Problem solving	Kang et al., 2021; Gavron et al., 2023	2	6	"Group art therapy aims to improve problem solving through art" (Kang et al., 2021)
Social bond and support		6	18	
Community building	Klorer, 1992; Carpendale, 2010; Wright et al., 2023	3	7	"… restoration work can function to work on building a sense of self and community" (Carpendale, 2010)
Group support and cohesion	Wright et al., 2023; Gavron et al., 2023	2	5	"… development of group cohesiveness" (Klorer, 1992)
Impact of group interaction	Wright et al., 2023	1	1	"Several members reported that the group prompted them to take up or renew other activities, including making more use of green spaces and with a changed perspective" (Wright et al., 2023)
Perspective sharing	Wright et al., 2023	1	1	"… how the surroundings prompted participants to look outside themselves" (Wright et al., 2023)
Relationship building skills	Peterson, 2015; Kang et al., 2021; Gavron et al., 2023	3	3	"… allowed for experiences and behaviours that are suitable for this developmental stage, such as building relationships, which encourage experiences of pleasure, responsibility, and at times frustration" (Gavron et al., 2023)
Trust development	Klorer, 1992	1	1	"… development of trust in others" (Klorer, 1992)
Self-discovery and personal growth		10	81	
Body-mind-environment awareness	Klorer, 1992; Carpendale, 2010; Peterson, 2015; Kopytin, 2021; Elkis-Abuhoff et al., 2022; Kang et al., 2021; Wright et al., 2023; Wardle, 2023; Gavron et al., 2023	9	37	"All of these activities focus on ways of making contact with the self and reintegrating disowned parts of the self. The focus is on renewing the capacity for wholeness and rebuilding a positive relationship to the environment" (Carpendale, 2010)<br>"Mindfulness-based arts therapeutic techniques can be integrated into ecotherapy practices. In this way body–mind–environment focused activities can support the goals of ecotherapy by fostering reconnection and returning to experiencing ourselves in the here and now as an embodied being" (Kopytin, 2021)

Themes / subthemes / codes	References	Files count	Text unit count	Example vignettes
Development of ecological identity	Klorer, 1992; Carpendale, 2010; Kopytin, 2021; Wright et al., 2023; Gavron et al., 2023	5	25	"A therapeutic perspective that considers the importance of the individual's ecological identity is based on a social constructivist view that the individual is constructed in relationship to their social and environmental context. This perspective does not separate the self from the world. Therapy should be considered in light of the world's situation and the individual's world view" (Carpendale, 2010)
Development of responsibility	Klorer, 1992; Gavron et al., 2023	2	2	"… allowed for experiences and behaviours that are suitable for this developmental stage, such as building relationships, which encourage experiences of pleasure, responsibility, and at times frustration" (Gavron et al., 2023)
Self-esteem enhancement	Klorer, 1992; Kang et al., 2021; Kopytin, 2021; Wright et al., 2023	4	7	"Among the sub-categories of self-esteem, there were statistically significant results in overall self-esteem and social self-esteem" (Kang et al., 2021)
Self-reflection	Peterson, 2015; Lee et al., 2020; Wright et al., 2023; Wardle, 2023	4	7	"The child presented in this paper engaged notably differently when sessions were taken outside. He began to explore and communicate more coherently about his life experiences and the death of his relative" (Wardle, 2023)
Sense of achievement	Gavron et al., 2023; Wright et al., 2023	2	3	"… a shared sense of achievement" (Gavron et al., 2023)
Trauma and grief management		4	8	
Grief and loss management	Carpendale, 2010; Wardle, 2023	2	3	"Restoration work focuses on the whole life cycle: birth, death, and transformation. Grief work, attending to the rites of passage and moving through the liminal stages" (Carpendale, 2010)
Trauma-informed care	Klorer, 1992; Gavron et al., 2023; Wardle, 2023	3	5	"His mother had requested Alex attend art therapy for emotional support to help cope and process multiple difficult life experiences and adversities. While Alex was on the waiting list for the service, he experienced a traumatic bereavement of a close family member who died by suicide" (Wardle, 2023)

Themes / subthemes / codes	References	Files count	Text unit count	Example vignettes
Creative self-expression		4	20	
Creative potential	Gavron et al., 2023; Wardle, 2023	2	8	"… using materials from nature is not necessarily seen as related to artistic talent or technique, so that there is a potential for self-expression without criticism which can support flow" (Gavron et al., 2023)
Flow state	Gavron et al., 2023	1	3	"The building phase was shown to facilitate an experience of flow" (Gavron et al., 2023)
Playfulness and enjoyment	Kang et al., 2021; Gavron et al., 2023; Wright et al., 2023; Wardle, 2023	4	9	"Art therapy related to forest activities includes and supports experiential learning and play and focuses on physical and sensory experiences, creative expression, and spontaneity in natural environments" (Kang et al., 2021)<br>"Some of the participants perceived tending the terrarium as a playful experience where mistakes were allowed: "I played with it a bit, once it seemed to have too much water" (Gavron et al., 2023)
Environmental sustainability		5	29	
Environmental awareness and knowledge	Steinhardt, 1998; Carpendale, 2010; Kopytin, 2021	3	13	"Most of the participants perceived choosing and planting the plants as the central experience in making the terrarium: "I treated it as a kind of a tiny garden. a garden in its own world". Some saw it as an ecosystem, whose goal was to survive: "I liked creating a system that would eventually be self-sufficient and last over time" (Elkis-Abuhoff et al., 2022)<br>"We can also help our clients enrich their ecological knowledge and develop their ecological consciousness and thus decrease their possible destructive impact on the natural environment and prevent risks of various physical and mental issues as a result of unsustainable ways of living and pathogenic environmental factors. We can do this, in particular, through waste reduction and an environmentally conscious attitude to our use of materials" (Kopytin, 2021)
Environmentally responsible behavior	Carpendale, 2010; Kopytin, 2021; Elkis-Abuhoff et al., 2022; Gavron et al., 2023	4	16	"The arts help people to feel in control of the environment and participate in its management and restoration. Art making can be used to promote individuals' and communities' active position in their relationship with the environment and develop their perception of themselves as people who are able to exert a certain amount of influence on it" (Kopytin, 2021)

Themes / subthemes / codes	References	Files count	Text unit count	Example vignettes
				“An art therapist can become environmentally conscious with regards to materials and use non-toxic materials, recycled materials and natural materials. Chalkboards can be used as reusable surfaces for conjoint art therapy activities like scribble tag” (Carpendale, 2010)
Nature engagement in nature-based art therapy		11	130	
Direct engagement		10	97	
Crafting with natural materials	Steinhardt, 1998; Carpendale, 2010; Kopytin, 2021; Kang et al., 2021; Elkis-Abuhoff et al., 2022; Gavron et al., 2023	6	14	“Creating your own sculpture using your own photos, branches, leaves, grass, and flowers" (Kang et al., 2021
Found object-driven self-reflection	Wardle, 2023; Wright et al., 2023	2	3	“While we were exploring between trees, crawling under branches and travelling to new areas, Alex came across a fire pit with blackened logs and ash. He asked me if I thought someone had been cremated there. We explored the idea together, playfully imagining different uses for a fire pit. We talked about cremation, but also making food, getting warm and talking around" (Wardle, 2023)
Miniature ecosystem building	Steinhardt, 1998; Elkis-Abuhoff et al., 2022; Gavron et al., 2023	3	8	“An expressive terrarium consists of a plant terrarium in a glass bowl that contains an ecosystem of living plants and objects reflecting the personal artistic-creative design of the maker" (Gavron et al., 2023)
Nature-focused photography	Peterson, 2015; Kang et al., 2021	2	2	“Walkabout integrates mindful outdoor walking with participants taking digital photographs followed by using their pictures to construct collages within an experiential framework of mindfulness and creativity that unfolds over 8 weeks" (Peterson, 2015)

Themes / subthemes / codes	References	Files count	Text unit count	Example vignettes
Outdoor mindful activities	Peterson, 2015; Kang et al., 2021; Kopytin, 2021; Wright et al., 2023; Wardle, 2023	5	10	“Sessions began with a mindfulness grounding exercise under a huge, ancient Plane tree. The group then chose a spot to walk towards, conducting individual check-ins along the way" (Wright et al.,2023)
Planting and caring for plants	Gavron et al., 2023	1	5	“Building the terrarium takes place in several stages and involves installing a bed of pebbles and netting, adding soil, and then choosing and placing the plants in the soil while designing the overall look of the terrarium. Found objects and art materials can be added as desired. The expressive terrarium innovates by combining nature-based and horticultural therapy with an art-based intervention” (Gavron et al., 2023)
Production of art materials from nature	Kang et al., 2021	1	1	“Making colored sand" (Kang et al., 2021)
Sensory interaction with natural elements	Steinhardt, 1998; Kang et al., 2021; Kopytin, 2021; Wright et al., 2023	4	5	“Feeling nature’s sounds, smells, touch, and light, and freely expressing these feelings" (Kang et al., 2021)
Wildlife exploration	Carpendale, 2010; Peterson, 2015; Wardle, 2023; Wright et al., 2023	4	7	“Quietly looking around, Alex became more aware of different animals, insects and plants he saw during our sessions. He started looking at the tops of trees to find squirrels and birds, and close at leaves and plants to point out slugs, snails and ants" (Wardle, 2023)
Indirect engagement		8	33	
Art-based exploration of ecological identity	Carpendale, 2010	1	1	“Ecological identity work can be done through exploring a sense of belonging to a place by using maps as a basis to create collage images. Maps can be used in a number of ways: a) to create memory maps to explore the memory of a special place from childhood; b) to explore cultural identity through making a map collage to represent and describe ancestry and family history; and

Themes / subthemes / codes	References	Files count	Text unit count	Example vignettes
				c) to explore a personal relationship to the environment. This could include a sacred or special place or a disturbed or violated place in the world" (Carpendale, 2010)
Art-based exploration of experiences in nature	Klorer, 1992	1	1	"All participants at some point during the summer, have to confront their fears and self-imposed boundaries. It may be a fear of caves, a fear of the dark, a fear of heights, fear of animals and unknown night creatures, real fears and fears of the imagination. In their journals, the children draw themselves in situations that may evoke their biggest fears. Yet, the drawings are executed from a position of strength, the children having just successfully confronted the fear" (Klorer, 1992)
Found object-driven self-reflection	Wardle, 2023; Wright et al., 2023	2	3	"While we were exploring between trees, crawling under branches and travelling to new areas, Alex came across a fire pit with blackened logs and ash. He asked me if I thought someone had been cremated there. We explored the idea together, playfully imagining different uses for a fire pit. We talked about cremation, but also making food, getting warm and talking around" (Wardle, 2023)
Landscape art creation	Peterson, 2015; Elkis-Abuhoff et al., 2022	2	2	"Participants were instructed to "think about a favorite place in nature that you enjoy visiting or would someday like to visit. Then create that landscape of nature" (Elkis-Abuhoff et al., 2022)
Use and creation of nature-inspired poetry	Carpendale, 2010; Wright et al., 2023	2	3	"At times the therapists would bring poems, works of art or non-fiction books to stimulate creative relating to nature" (Wright et al.,2023)
Use of analogies in verbal reflection	Carpendale, 2010; Wardle, 2023: Wright et al., 2023	3	6	"He started looking at the tops of trees to find squirrels and birds, and close at leaves and plants to point out slugs, snails and ants. Additionally, he started to bring up the topics of life, birth and death. He would talk about the insects and animals being born as well as pointing out parts of plants that had died" (Wardle, 2023)
Use of nature-inspired color palette	Steinhardt, 1998; Elkis-Abuhoff et al., 2022	2	2	"… the use of both materials and colors found to be prevalent within nature can also aid art therapists in utilizing a nature-based approach in sessions, even if the participants are restricted to an indoor setting" (Elkis-Abuhoff et al., 2022)

Themes / subthemes / codes	References	Files count	Text unit count	Example vignettes
Use of nature-inspired metaphors	Steinhardt, 1998; Carpendale, 2010; Elkis-Abuhoff et al., 2022; Gavron et al., 2023; Wright et al., 2023; Wardle, 2023	6	15	"Water changes form in response to temperature and gravity by evaporating, freezing, or flowing, states which may be emotional metaphors. Water reflects what is not part of it, supports what is on it, and transparently reveals what is within it. Water cleanses and cools but also pours down in wrath from the sky, flooding and drowning" (Steinhardt, 1998)
Work with nature photography	Carpendale, 2010; Peterson, 2015	2	3	"Each selects 10 photos for printing in a variety of formatted sizes; selected photos, made available in the following session, are printed on matte photo paper, which is amenable to use with a range of art and collage media" (Peterson, 2015)
Core elements of nature-based art therapy		10	94	
Artwork		9	47	
Digital art	Peterson, 2015; Kang et al., 2021	2	2	"Participants are asked to take pictures of what they move toward as pleasant and to also photograph what they experience as unpleasant" (Peterson, 2015)
Environmental art	Kang et al., 2021; Gavron et al., 2023	2	7	"Making a house using cloth and wood with the group members" (Kang et al., 2021)
Literary art	Carpendale, 2010; Wright et al., 2023	2	6	"The group uses mindfulness and grounding exercises, poetry, stories, art making, and group discussion in a natural setting to enhance member's sense of closeness to nature, to build confidence in relating to others and to discover new tools for enhanced well-being in their lives" (Wright et al., 2023)
Visual art	Klorer, 1992; Steinhardt, 1998; Carpendale, 2010; Peterson, 2015; Kang et al., 2021;	8	31	"... the children continue to draw and paint their summer experiences" (Klorer, 1992)

Themes / subthemes / codes	References	Files count	Text unit count	Example vignettes
	Elkis-Abuhoff et al., 2022; Wright et al., 2023; Wardle, 2023; Gavron et al., 2023			
Materials		9	26	
Conventional art materials	Elkis-Abuhoff et al., 2022	6	6	“Participants in the drawing group were presented with 11× 14 white paper, medium tip markers and colored pencils" (Elkis-Abuhoff et al., 2022)
Earth-based materials	Steinhardt, 1998; Carpendale, 2010; Kang et al., 2021; Elkis-Abuhoff et al., 2022; Gavron et al., 2023; Wright et al., 2023	6	9	“Participants could then choose what do; drawing, collage, collecting natural materials, using clay, making prints" (Wright et al.,2023)<br>“Sand is a formless collection of granules, shifting indifferently in response to external forces. Sand combined with water enables one to build strong structures, smooth or texture its surface, enter into its depths, bury and uncover, or make lacy, fantasy drip-castles. Particular forms of play are elicited by the nature of sand. At the beach the entire body is often involved. Sandplay in the therapy room is symbolic and nonverbal and uses the hands only. Here sand can become the formlessness before creation, while client and therapist witness the bringing of forms into the world" (Steinhardt, 1998)
Found human-made objects	Wardle, 2023	1	1	“He collected materials he found and arranged these in a circle. They included a variety of naturally occurring materials such as rocks and branches as well as waste people left behind such as beer bottle caps, clothing and food packaging" (Wardle, 2023)
Plant-based materials	Kang et al., 2021; Kopytin, 2021; Elkis-Abuhoff et al., 2022. Gavron et al., 2023. Wardle, 2023	5	7	“In the collage group, participants were provided with nature-based collage items, such as flower petals, leaves, sticks, and pebbles” (Elkis-Abuhoff et al., 2022)

Themes / subthemes / codes	References	Files count	Text unit count	Example vignettes
Water-based materials	Steinhardt, 1998; Carpendale, 2010	2	3	"This could include sand or snow sculptures, and drawings or mandalas" (Carpendale, 2010)
Therapy settings		8	21	
Indoor therapy room	Gavron et al., 2023	1	1	"This study constitutes a first step toward examining an art and nature-based intervention that combines therapeutic horticulture and nature-based ecological art therapy. The expressive terrarium engaged the participants in the use of multiple materials in a complex ongoing creative process that invited nature indoors into a personal creative and playful space" (Gavron et al., 2023)
Back yard	Peterson, 2015	1	1	"… experiential reflections included a deep appreciation for being with other persons with cancer in a setting outside the chemo center, an opportunity she had not experienced" (Peterson, 2015)
Forest	Kang et al., 2021	1	3	"During the program simulation, art therapists explored the forest terrain and natural objects that could be used during the program" (Kang et al., 2021)
Garden	Kopytin, 2021; Gavron et al. 2023; Wardle, 2023	3	4	"The reserve has a woodland area, a meadow area and a walled garden" (Wardle, 2023)
Greenhouse	Gavron et al., 2023	1	5	"After making the terrarium and placing it in the college greenhouse, the students were asked to tend it at least once a week. This included watering or aerating the jar if necessary and monitoring the development and changes inside the terrarium such as mold, decay or need for pruning" (Gavron et al., 2023)
Meadow	Wardle, 2023	1	1	"The reserve has a woodland area, a meadow area and a walled garden" (Wardle, 2023)
Mountain	Kang et al., 2021	1	1	"For 10 min after starting the session, the participants were asked to find a place on the mountain where they could work" (Kang et al., 2021)
Park	Wright et al., 2023; Wardle, 2023	2	6	"The reserve has a woodland area, a meadow area and a walled garden. While it is common to pass a few people, the reserve is quiet for a city park" (Wardle, 2023)
Woodland	Wardle, 2023	1	1	"The reserve has a woodland area, a meadow area and a walled garden" (Wardle, 2023)

Themes / subthemes / codes	References	Files count	Text unit count	Example vignettes
Challenges in nature-based art therapy		5	44	
Complexities in client assistance		2	8	
Session planning	Wright et al., 2023	1	1	“The therapists spoke about the importance of the structure of the sessions in regulating anxiety and promoting safe and productive engagement. The combination of meeting at the same time, in the same place, having a moment to pause, mindfulness exercise, then the exploration where members could go off on their own if they wanted, the return to reflect together was felt to be very holding. Therapists adapted the model to enable them to give a clear commitment to service users" (Wright et al., 2023)
Client engagement	Wright et al., 2023	1	2	
Group dynamics	Wright et al., 2023	1	3	“The therapists experienced complexities around balancing group composition and managing group dynamics in an open space” (Wright et al., 2023)
Location and schedule management	Wright et al., 2023	1	1	“Judging how long to wait at the start of sessions at a rendezvous point in the open could be challenging if members did not arrive on time or where there was uncertainty as to whether they weren’t coming. Some members were coming long distances so therapists didn’t want to leave late comers behind” (Wright et al., 2023)
Privacy management	Wardle, 2023	1	1	“Finally, it would also be helpful to examine the impact of outdoor locations on the therapeutic process and how to best overcome the challenges in running services outdoors such as time and travel, privacy, access to suitable outdoor spaces, weather, hours of daylight and access to outdoor clothing such as rain jackets and walking shoes” (Wardle, 2023)
Constraints in nature access		5	26	
Budget constraints	Wardle, 2023	1	1	“Initial sessions could not take place outdoors as outdoor sessions can take twice the time when accommodating travel which was not possible initially due to service quotas” (Wardle, 2023)
Institution-related constraints	Elkis-Abuhoff et al., 2022	1	1	“… such as prison, inpatient psychiatry, memory-care units, and within the clean environment of a medical setting” (Elkis-Abuhoff et al., 2022)

Themes / subthemes / codes	References	Files count	Text unit count	Example vignettes
Time and travel constraints	Lee et al., 2020; Kang et al., 2021; Wright et al., 2023; Wardle, 2023	4	6	"Finally, it would also be helpful to examine the impact of outdoor locations on the therapeutic process and how to best overcome the challenges in running services outdoors such as time and travel" (Wardle, 2023)
Physical mobility-related constraints	Elkis-Abuhoff et al., 2022; Wardle, 2023	2	2	"We arranged for Alex to meet me at the community centre at the start of the session. We travelled together to and from the nature reserve by taxi. This allowed for safe and reliable transportation which also worked to meet the caring needs of Alex's family member who had multiple medical conditions. It also allowed for the option to meet indoors to check if Alex was still okay to work outdoors" (Wardle, 2023)
Urban nature access constraints	Elkis-Abuhoff et al., 2022	1	4	"These factors were further considered with the global lockdown orders that took place with the Covid-19 pandemic in 2020 where some individuals were unable to access nature depending on their location" (Elkis-Abuhoff et al., 2022)
Psychosocial and cultural barriers		8	49	
Multicultural perspectives on nature engagement	Carpendale, 2010; Kopytin, 2021; Elkis-Abuhoff et al., 2022; Gavron et al., 2023; Wardle, 2023	5	6	"A therapeutic perspective that considers the importance of the individual's ecological identity is based on a social constructivist view that the individual is constructed in relationship to their social and environmental context. This perspective does not separate the self from the world. Therapy should be considered in light of the world's situation and the individual's world view" (Carpendale, 2010)<br>"Some of the participants experienced frustration with the physical distance: "I didn't feel a connection taking care of it, I guess because it wasn't at my house, here at home it's more accessible, it's on the table and I have a much deeper connection to it". Another participant felt that tending the terrarium created an emotional distance: "I felt like having another commitment beyond what I have; now that it is at home, once in a while I water it and I feel good in its presence" (Gavron et al., 2023)
Levels of nature connection	Carpendale, 2010; Peterson, 2015; Lee et al., 2020;	7	41	"Ecopoiesis is a quality and mechanism of the coevolution of the human being and nature, a conscious and responsible cocreation of humankind with the natural world, based on its physical, emotional, and spiritual connection with it" (Kopytin, 2021)

Themes / subthemes / codes	References	Files count	Text unit count	Example vignettes
	Kopytin, 2021; Elkis-Abuhoff et al., 2022; Gavron et al., 2023; Wright et al., 2023			"Disruptions in one's ability to connect with, and experience, nature have been found to contribute to declines in both mental health and emotional wellness" (Carpendale, 2010)<br>"It posits that distancing oneself from nature has a negative effect on mental health and suggests that strengthening one's connections with nature is likely to support mental health by reducing the symptomatology of disorders" (Gavron et al., 2023)
Eco-anxiety and environmental grief	Carpendale, 2010	1	2	"… underlying feelings of guilt, fear, rage, grief, which are all part of environmental despair. There can be guilt about the threatening environmental catastrophe in that one hasn't taken enough action to prevent it. There are fears of both personal suffering and fear of witnessing the suffering of loves ones and others" (Carpendale, 2010)
Risk management		3	10	
Emergency preparedness	Wardle, 2023	1	2	"For the sessions, I brought art material, a sketchbook, water, a first aid kit and a mobile phone. An emergency contact was assigned at the charity in case an incident was to happen during the session" (Wardle, 2023)
Equipment requirements	Wardle, 2023	1	1	"Finally, it would also be helpful to examine the impact of outdoor locations on the therapeutic process and how to best overcome the challenges in running services outdoors such as time and travel, privacy, access to suitable outdoor spaces, weather, hours of daylight and access to outdoor clothing such as rain jackets and walking shoes" (Wardle, 2023)
Management of physical comfort and needs	Wardle, 2023	1	1	"It also allowed for the option to meet indoors to check if Alex was still okay to work outdoors and ready for sessions as there were no shelters or toilets available at the reserve" (Wardle, 2023)
Outdoor safety training	Kang et al., 2021	1	1	"… experimental group received basic forest safety training before the program started. Forest safety education aimed to introduce safety rules that will help participants" (Kang et al., 2021)
Unpredictable or inclement weather	Kang et al., 2021; Wright et al., 2023; Wardle, 2023	3	5	"In addition, it was difficult to run the program on a set date and time due to weather changes, such as fine dust and heavy rain" (Kang et al., 2021)
